# Field evaluation of hermetic and synthetic pesticide-based technologies in smallholder sorghum grain storage in hot and arid climates

**DOI:** 10.1038/s41598-021-83086-3

**Published:** 2021-02-12

**Authors:** Macdonald Mubayiwa, Brighton M. Mvumi, Tanya Stathers, Shaw Mlambo, Tinashe Nyabako

**Affiliations:** 1 Department of Agricultural and Biosystems Engineering, Faculty of Agriculture, Environment and Food Systems, University of Zimbabwe, P. O. Box MP 167, Mt Pleasant, Harare, Zimbabwe; 2 Natural Resources Institute (NRI), University of Greenwich, Central Avenue, Chatham Maritime, Kent, ME4 4TB UK

**Keywords:** Non-model organisms, Entomology

## Abstract

Field evaluation of six grain storage technologies under hot and arid conditions (32–42 °C; rainfall < 450 mm/year) in two locations in Zimbabwe were conducted over two storage seasons. The treatments included three hermetic technologies (Purdue Improved Crop Storage bags, GrainPro Super Grainbags, metal silos); three synthetic pesticide-based treatments; and an untreated control, all using threshed sorghum grain. Sampling was at eight-week intervals for 32 weeks. Highly significant differences (p < 0.01) occurred between hermetic and non-hermetic treatments regarding grain damage, weight loss, insect pest populations, and grain moisture content; with the hermetic containers exhibiting superior grain protection. Weight losses were low (< 3%) in hermetic treatments compared to pesticide-based treatments (3.7 to 14.2%). *Tribolium castaneum* developed in metal silos, deltamethrin-incorporated polypropylene bags and a pesticide treatment containing deltamethrin 0.13% and fenitrothion 1% while *Sitotroga cerealella* developed in a pesticide treatment containing pirimiphos-methyl 0.16% + thiamethoxam 0.036%. Mechanisms of survival and development of these pests in the tested treatments and under similar climatic conditions need further elucidation. These hermetic technologies can be successfully used by smallholder farmers in developing countries as alternatives to synthetic pesticides for protecting stored-sorghum grain under hot and arid climatic conditions to attain household food security. To our knowledge, this is the first published study on modern hermetic storage of sorghum grain under typical smallholder storage conditions and involving stakeholders.

## Introduction

Projections suggest that food demand in sub-Saharan Africa (SSA) will triple from 2014 levels to meet the anticipated doubling of the region’s population to around 2.1 billion people by 2050^1,2^. However, this will be challenging to achieve as in many parts of SSA, crops can only be harvested once per year and may fail when prevailing climatic and edaphic conditions are unfavourable, a situation likely to be exacerbated by climate change and increasing climate variability. Climate change is expected to expose the rapidly growing SSA populations to increased food and nutrition insecurity^3^. In response to climate change and increasing climate variability, characterised by droughts and inadequate rainfall, farmers have scaled-up their production of small grains^4^, particularly sorghum and millets^5^. Of these, sorghum has a wider production and distribution range due to its higher yield per hectare compared to finger and pearl millets. In addition to being drought and heat tolerant, sorghum has temporary tolerance to waterlogging, making it ideal in drought-prone areas; it can tolerate intermittent floods and can do well in saline and sodic soils^5–7^. Despite the relatively higher yields compared to the other small grains, a high proportion of the sorghum produced is then lost postharvest. Evidence-based estimates suggest approximately 12% of Zimbabwe’s sorghum is lost annually along the post-production chain^8^. This translates to over 9,400 tonnes in 2018 with a monetary value of approximately US$3.7 million using Zimbabwe Grain Marketing Board prices. There has been limited efforts to address these sorghum postharvest losses in Africa^9,10^. Practical postharvest grain storage options need to be developed to complement existing crop production efforts to achieve food and nutrition security.

Synthetic pesticides have been one of the major means of protecting stored grain against insect pests^11^. However, in SSA such pesticides are sometimes unavailable, expensive and/or adulterated^12,13^. The efficacy of these insecticides is also greatly influenced by environmental conditions, particularly temperature and relative humidity^11,14^, dosage rates and the dominant insect pest species. Climate change points to a warming trend and highly variable rainfall patterns^15^, presenting new problems to postharvest grain handling through possible altered pest physiology, spectrum, behaviour and pesticide efficacy.

Hermetic storage technologies are gaining momentum in grain storage and are known to be effective in providing long-term, chemical-free and sustainable grain storage for several grain crops over extended storage seasons^16–21^. These technologies work on the principle of creating a modified environment within the storage container, and are constructed of materials with very low oxygen permeability^21^. Respiration by biological agents such as insects, mites, microflora and the grain itself within the storage facility will deplete the oxygen and cause a build-up of carbon dioxide, suffocating any pests that might be present^18,22,23^. The growing demand for pesticide-free and insect pest-free food products highlights the need for researchers and farmers to evaluate pesticide-free hermetic storage technologies^24^ as alternative options to synthetic chemical grain storage pesticides.

Non-chemical technologies can be an attractive option as they have no known negative effects on human and animal health. Recently introduced hermetic bags, and metal silos offer farmers pesticide-free grain storage options against insect pests^18^. Metal silos are cylindrical containers which can be tailor-made to fit the farmer’s capacity and circumstances (indoor or outdoor), they are fabricated from galvanized iron, with sealable inlet and outlet valves that help prevent entry of oxygen from the outside environment^25^. A lit candle is placed inside the container immediately prior to closing it, purported to deplete oxygen and enhance insect kill^26^. Hermetic bags have one or, in some cases, two high density polyethylene liners that are placed inside the outer polypropylene bag. Each bag is securely sealed to effect insect suffocation within the stored grain. Among other modern technologies are pesticide-incorporated polypropylene bags which kill storage insects that come into contact with the fabric and minimise the amount of pesticide in contact with the grain. These technologies have not been adequately tested for efficacy under extreme conditions (hot and dry), particularly for the storage of small grains such as sorghum; crops known to be climate-resilient. The objective of the current study was to evaluate the efficacy of a range of hermetic grain storage technologies compared to synthetic pesticide-based treatments on sorghum grain stored under hot and arid conditions.

## Materials and methods

### Site description

**Figure 1:**
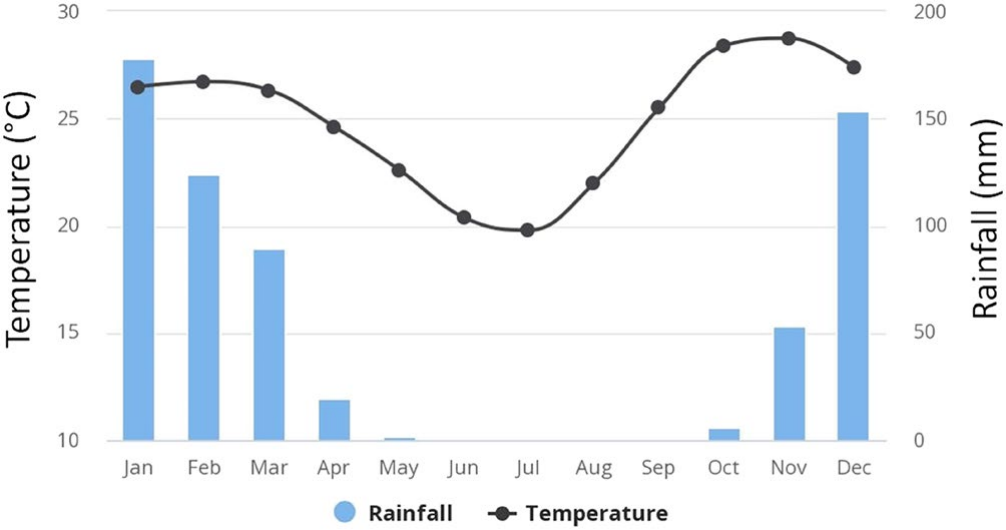
Average monthly temperature and rainfall at location 16°23′ S, 30° 58 E in Mbire district of Zimbabwe for 1991–2016. *Source*: World Bank Group Climate Change Knowledge Portal, 2021.

Field experiments to evaluate different grain storage technologies were carried out in two wards of Mbire District (Ward 8 and 15) (a ward is the second level administrative unit after the district) in the northern part of Zimbabwe (16°10′0.60" S, 30°34′14.99" E). Mbire district lies in the Zambezi valley, and experiences high mean annual temperature of approximately 25 °C, with summer temperatures as high as 42 °C. The area receives low mean annual rainfall, generally below 450 mm per year (Fig. 1)^27^.

### Experimental design and treatments

Six different grain storage technologies and an untreated control were evaluated in a randomised complete block design with four blocks of each of the seven treatments per ward, during two storage seasons (2014/15 and 2015/16). The blocks also constituted the replicates. The treatments included hermetic technologies, synthetic pesticide-based treatments and an untreated control (Table 1). Two brands of hermetic bags were used—PICS and Super Grainbag. PICS bags consist of two inner liner bags each made of 80 microns thick high-density polyethylene (HDPE), details of the oxygen transmission rate were not available from the manufacturer. This bag was manufactured by Polypack Limited (Maselema, Blantyre, Malawi) under franchise from Purdue University (West Lafayette, Indiana, USA). The Super Grainbag IV-R was manufactured by GrainPro Inc. (Zambales, Philippines) and has just one inner liner bag of 78 microns thick plastic with an oxygen transmission rate of < 50 cc/m^2^ per day at 0.1 MPa and water vapour transmission rate of < 10 g/m^2^ per day^28^. This liner is then placed inside a polypropylene bag. The plastic liners were tested for perforations or air-leakages by inflating the bags, closing and compressing them^29^. The ZeroFly storage bag is a polypropylene bag containing deltamethrin incorporated into the bag fabric at 3 mg/kg by the manufacturer (Vestergaard, Lausanne, Switzerland). A metal silo was included as part of the hermetic treatments. It is a cylindrical container with an inlet and an outlet that are used for loading and offloading the grain. The silos were placed on top of wooden pallets, and their inlets and outlets were sealed off using strips of rubber bands measuring 1 m long by 5–6 cm wide. A lit candle was enclosed to hasten oxygen depletion after loading the sorghum grain.

**Table 1:** List of treatments used in Ward 8 and 15 of Mbire district, Zimbabwe during the sorghum storage trials in the 2014/15 (Year 1) and 2015/16 (Year 2) storage seasons.

Treatment group	Treatment name	Application rate per 50 kg of grain	Year 1	Year 2
Hermetic technologies	Metal silo	No pesticide	✓	✓
	Purdue Improved Crop Storage (PICS) bag	No pesticide	✓	✓
	GrainPro Super Grainbag	No pesticide	✓	✓
Synthetic pesticide-based	Pesticide 1 (Shumba Super dust)—Mbire	25 g	✓	✓
	Pesticide 1 (Shumba Super dust)—Harare	25 g	✓	-
	ZeroFly storage bag (pesticide incorporated bag)	N/A	✓	✓
	Pesticide 2 (Actellic Gold dust)	25 g	-	✓
Untreated	Negative control	N/A	✓	✓

‘–’ indicates treatment not included in that year; N/A = not applicable.

Shumba Super dust = fenitrothion 1% + deltamethrin 0.13%; Actellic Gold dust = pirimiphos-methyl 0.16% + thiamethoxam 0.036%; ZeroFly storage bag = deltamethrin-incorporated polypropylene bag at 3 mg/kg.

Two treatments of the synthetic pesticide Shumba Super dust were included as farmers alleged that when they bought the pesticide from their local shops, it was not effective, hence a comparison of the product bought locally from an agro-dealer and the same product bought from a large agro-dealer in the capital city, Harare, was included in the experiment. The pesticide is made up of 1% fenitrothion (organophosphate) and 0.13% deltamethrin (pyrethroid). Untreated grain in polypropylene bags was used as a negative control in both seasons.

Due to the absence of significant differences between the two Shumba Super dust pesticides during the first season, the locally bought Shumba Super dust treatment was replaced by Actellic Gold dust pesticide in the 2015/16 season. Actellic Gold dust is a recently introduced synthetic storage pesticide on the Zimbabwean market. It contains 0.16% pirimiphos-methyl (organophosphate) and 0.036% thiamethoxam (nionicotinoid).

Treatment preparation and application was done in one central place, and the treatment materials were allocated to 50 kg lots of freshly harvested and dried sorghum grain in each ward. The sorghum grain variety SC Sila was used in the first season of the experiment while in the second season a mixture of SC Sila and Macia varieties (≈ 1:1) was used because insufficient quantities of a single variety were available. Smallholder farmers in SSA tend to store their sorghum grain intended for consumption as mixed varieties, while for seed use, sorghum varieties are more commonly stored separately, so sorghum grain storage technologies need to be efficacious on a wide range of varieties and grain mixtures. The grain used in the experiment had not been subjected to any pest control treatments nor fumigation before being used in the experiment. All the grain was thoroughly mixed before set-up to homogenise any existing damage or insect populations in it. All synthetic pesticides were applied at label rates of 25 g of pesticide dust to 50 kg of grain (0.05% w/w), and were shifted at least three times during admixing, to ensure even distribution of the pesticide during setting-up of the experiments. At set-up and subsequently after every eight weeks, 500 g samples (approximately 25,000 grains) were collected from each treatment using a multi-compartmented grain probe, taking special care not to perforate the hermetic bags. After sampling from pesticide treatments, the grain probes were washed using water and a detergent, and were dried before proceeding to other treatments to avoid cross-contamination by pesticides. No artificial addition of live insect pests was done and the experiments were therefore dependent on natural pest infestation activity.

The experiments were housed inside timber-walled and termite-mound-soil-plastered, compartmentalized granaries at four households in each of the two wards. Easylog data loggers (Model EL-USB-1, Whiteparish, Wiltshire, SP5 2SJ, United Kingdom) were installed 1.5 m above the ground in the storage rooms to measure temperature and relative humidity data every 30 min throughout the study. The granaries were grass-thatched to provide a cool environment for the treatments in-store. The households who hosted the experiment were selected jointly by participating farmers’ groups and agricultural extension staff based on accessibility, store design uniformity and security of the stores in each participating ward. Each household would act as a Learning Centre, where local farmers, extension staff and researchers would meet during sampling and undertake the sampling and observation tasks together, which provided opportunities for discussion of the results and other pertinent postharvest issues.

### Data collection and analyses

After sampling, the samples were taken to the laboratory at the University of Zimbabwe where they were weighed, sieved to remove the chaff, which was also weighed and expressed as percentage weight per kilogram. After sieving, the total number of live and dead insects were counted by species and expressed as a proportion of a kilogram grain sample. Dead insects were confirmed by dorsally prodding their abdomen using some soft brushes^30^. This was followed by grain moisture content measurement using a pre-calibrated Dickey-John digital moisture meter (M3G™ model, Dickey-John Corporation, Minneapolis, USA). A riffle divider was used to divide grain samples into eight parts, of which three parts (three-eighths) were each analyzed for grain damage percentage using the formula:

$$Grain\,damage\,\% = \left( {\frac{Nd}{{Nd + Nu}}} \right)*100$$

where N_d_ = number of insect damaged grains, N_u_ is the number of undamaged grains.

Each sub-sample of sorghum grain used for damage assessment weighed approximately 190 g. Grain rotting was expressed as the proportion of rotten kernels to total number of kernels in the sample, while grain weight loss percentage was calculated using the Count and Weigh method^31^ based on the formula:

$$Weight\, loss\,\% = \frac{{\left( {Wu*Nd} \right) - \left( {Wd*Nu} \right)}}{{Wu\left( {Nu + Nd} \right)}}*100$$

where N_d_ = number of damaged grains, N_u_ = number of undamaged grains, W_d_ = weight of damaged grains, W_u_ = weight of undamaged grains.

Data on the total number of adult live insects and mean grain moisture content were presented graphically in MS Excel. The data on total number of live insects and total insects (live and dead) were correlated with insect grain damage, grain weight loss and moisture content. The data were also subjected to rANOVA analysis after log_10_ (x + 1) transformation, where **x** is the number of insects per treatment, per sampling time^32^.

Grain damage, grain weight loss and rotten grain data were subjected to repeated measures analysis of variance (rANOVA) in Genstat 14^th^ edition following tests for conformity with ANOVA assumptions. The rANOVA was selected as sampling was carried out from the same experimental unit over the entire storage period. Where significant differences were found, separation of treatment means was done using Tukey’s test, while that for storage time (sampling periods) was done using Fisher’s Protected LSD test. Some sets of the percentage grain weight loss data did not meet the assumptions of ANOVA, and were square-root transformed before being analysed^33^.

Weather data from the data-loggers were downloaded every 8 weeks. Mean maximum and minimum temperatures were calculated by averaging the daily maximum or minimum temperatures for every four-week period throughout the entire storage season. Four-weekly mean temperatures were also calculated. These were used to plot visual charts in MS Excel.

### Ethical approval

This article does not contain any studies with animals performed by any of the authors.

### Informed consent

For the farmers who hosted the trials and participated in studying the efficacy performance of the grain storage technologies, informed consent was obtained prior to the study.

## Results

### Insect grain damage

**Figure 2:**
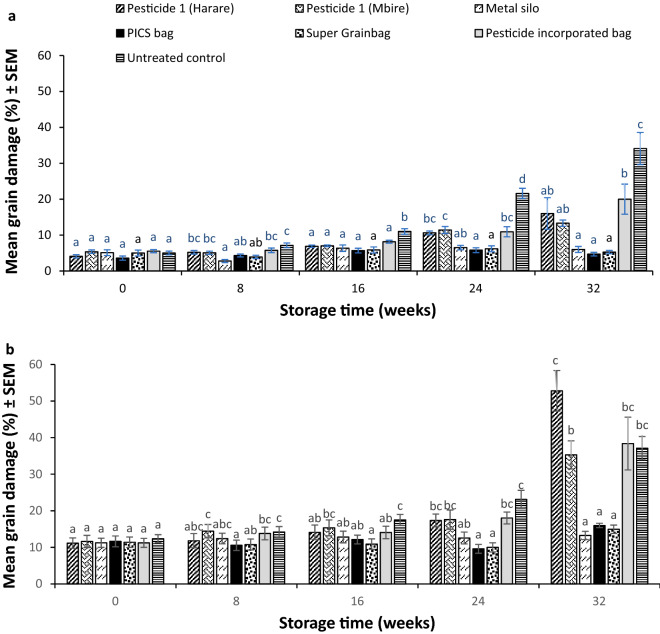
The effect of different grain storage treatments on sorghum grain insect damage (% ± SEM) during: (a) the 2014/15, and (b) the 2015/16 storage seasons in Mbire district, Zimbabwe (n = 8). Pesticide 1 = fenitrothion 1% + deltamethrin 0.13%; Pesticide 2 = pirimiphos-methyl 0.16% + thiamethoxam 0.036%; Pesticide-incorporated bag = deltamethrin-incorporated polypropylene bag at 3 mg/kg. Means were compared and separated at each sampling period (storage time) using Tukey’s test at 0.05 level.

Overall treatment effects were significant with regards to grain damage during the 2014/15 (F_6, 266_ = 36.28; p < 0.01) and 2015/16 (F_6, 266_ = 42.01; p < 0.01) storage seasons (Table 2). The hermetic storage technologies (metal silo and the two hermetic bags) were most effective in maintaining low grain damage due to insect pests during the two storage seasons (Fig. 2a,b). High grain damage was recorded in the untreated control (34 and 37%), deltamethrin-incorporated polypropylene bag (20 and 38%) and Pesticide 1 (a pesticide containing fenitrothion and deltamethrin) (16 and 52%) in 2014/15 and 2015/16 storage seasons, respectively. There were no significant differences in the performance of Pesticide 1 when obtained from two different sources (from a registered Harare agro-dealer and the other from an agro-dealer in Mbire district) during the first storage season (Fig. 2a). Overall, initial grain damage levels were high in the second season (15.3%) as compared to the first season (4.7%).

**Table 2:** Overall treatment effects on sorghum grain damage over the 32-week storage periods in Mbire district, Zimbabwe during the 2014/15 and 2015/16 storage seasons (n = 40).

Treatment	2014/15 season	2015/16 season
Pesticide 1 (Harare)	8.6 ± 1.10b	21.4 ± 2.83c
Pesticide 1 (Mbire)	8.4 ± 0.60b	–
Pesticide 2	–	18.8 ± 1.73bc
Metal Silo	5.4 ± 0.38a	12.5 ± 0.61ab
PICS bag	4.8 ± 0.27a	12.0 ± 0.62a
Super Grainbag	5.2 ± 0.33a	11.6 ± 0.64a
Pesticide-incorporated bag	10.1 ± 1.21b	19.1 ± 2.18bc
Untreated control	15.8 ± 1.96c	21.0 ± 1.69c
P-value	< .001	< . 001
F_2, 266_	36.28	42.01

Means are compared per column and those which are followed by different letters are statistically different from one another using Tukey’s test at 0.05 level.

‘–’ indicates treatment was excluded.

During the 2015/16 storage season, high grain damage levels were recorded in grain treated with a newly introduced Pesticide 2 (containing pirimiphos methyl and thiamethoxam) (~ 35%) (Fig. 2b). The damaged grains were heavily webbed and clustered together by moth activity.

Storage time had a significant effect on overall grain damage during the 2014/15 (F_4, 266_ = 54.82; p < 0.01) and 2015/16 (F_4, 266_ = 42.01) storage seasons (Table 3). Significant increases in grain damage and grain weight losses were recorded from 16 weeks storage in the two seasons. Treatment * time interactions were significant in both seasons.

**Table 3:** Overall time effects on mean percentage grain damage in Mbire district, Zimbabwe during the 2014/15 and 2015/16 sorghum storage seasons (n = 56).

Storage time (weeks)	2014/15 season	2015/16 season
0	4.8 ± 0.23a	11.5 ± 0.50a
8	4.9 ± 0.25a	12.5 ± 0.62ab
16	7.3 ± 0.33b	13.0 ± 0.66ab
24	10.4 ± 0.77c	15.5 ± 0.91b
32	14.2 ± 1.68d	29.7 ± 0.36c
P-value	< .001	< .001
F-value	54.82	42.01

Means are compared per column, and those which do not share the same letter are significantly different from one another using Fishers Protected LSD test at 0.05 level.

### Insect grain weight loss

Grain weight loss was high in the untreated control (up to 13.8% and 8.2% by the end of the 2014/15 and 2015/16 storage seasons, respectively), in a synthetic pesticide containing fenitrothion and deltamethrin (14.0% during the 2015/16 storage season) and in the ZeroFly pesticide-incorporated storage bag (10.8% and 10.5% during the 2014/15 and 2015/16 storage seasons, respectively). The least grain weight loss occurred in all the three hermetic treatments during both storage seasons (Table 4). The overall treatment effects on grain weight loss were significant (F_6, 266_ = 18.01; p < 0.01 and F_6, 266_ = 6.7; p < 0.01) (Table 5) as well as those for storage times (F_4, 266_ = 45.10; p < 0.01 and F_4, 266_ = 33.42; p < 0.01) (Table 6) during the first and second seasons, respectively. The treatment-time interactions were also significant (F_24, 150_ = 8.83; p < 0.01 and F_24, 150_ = 6.63; p < 0.01) during the 2014/15 and 2015/16 storage seasons, respectively.

**Table 4:** Mean percentage sorghum grain weight losses at selected critical points during the two storage seasons in Mbire district, Zimbabwe (n = 8).

Treatment	2014/15 storage season			2015/16 storage season		
	0 weeks	24 weeks	32 weeks	0 weeks	24 weeks^Ϯ^	32 weeks
Pesticide 1 (Harare)	0.7 ± 0.15	3.3 ± 0.49bc	3.7 ± 1.17ab	1.5 ± 0.16	3.5 ± 0.33 cd	14.0 ± 1.65c
Pesticide 1 (Mbire)	0.7 ± 0.28	5.0 ± 1.42 cd	5.9 ± 1.04b	–	–	–
Pesticide 2	–	–	–	2.0 ± 0.27	2.9 ± 0.76bc	7.7 ± 1.2b
Metal silo	0.5 ± 0.16	1.4 ± 0.10ab	1.4 ± 0.10a	1.4 ± 0.21	1.5 ± 0.19ab	2.5 ± 0.33a
PICS bags	0.4 ± 0.11	1.0 ± 0.27a	1.1 ± 0.27a	1.7 ± 0.21	0.8 ± 0.08a	2.7 ± 0.49a
Super Grainbag	1.0 ± 0.42	1.1 ± 0.18a	1.5 ± 0.48ab	1.8 ± 0.22	0.9 ± 0.10a	2.8 ± 0.49a
Pesticide-incorporated bag	0.9 ± 0.33	2.6 ± 0.63ab	10.8 ± 1.67c	1.4 ± 0.09	3.7 ± 0.65 cd	10.5 ± 2.52bc
Untreated control	0.6 ± 0.19	5.7 ± 0.89d	13.8 ± 3.08c	1.7 ± 0.19	4.6 ± 0.88d	8.2 ± 1.54b
P-value	0.73	< 0.01	< 0.01	0.234	< 0.01	< 0.01
F-value	0.60	7.33	13.12	1.41	8.24	10.19

^Ϯ^Mean separation letters in this column were derived from square root transformed data; ‘–’ indicates absence of treatment in the ward (s); Means were compared per column, and those which do not share the same letter are significantly different from one another using Tukey’s test at 0.05 level. Pesticide 1 = fenitrothion 1% + deltamethrin 0.13%; Pesticide 2 = pirimiphos-methyl 0.16% + thiamethoxam 0.036%; Pesticide-incorporated bag = deltamethrin-incorporated polypropylene bag at 3 mg/kg.

**Table 5:** Overall treatment effects on sorghum grain weight loss during the 2014/15 and 2015/16 storage seasons in Mbire district, Zimbabwe (n = 40).

Treatment	2014/15 season	2015/16 season
Pesticide 1 (Harare)	2.0 ± 0.32b	4.76 ± 0.83b
Pesticide 1 (Mbire)	3.1 ± 0.49c	–
Pesticide 2	–	3.5 ± 0.43ab
Metal Silo	1.3 ± 0.12ab	1.8 ± 0.13a
PICS bag	1.0 ± 0.12a	1.6 ± 0.16a
Super Grainbag	1.3 ± 0.18ab	1.7 ± 0.17a
Pesticide-incorporated bag	3.6 ± 0.70c	4.1 ± 0.54b
Untreated control	4.6 ± 1.00d	4.0 ± 0.74b
P-value	< .001	< .001
F_-_value	16.27	6.70

Means are compared per column, and those which do not share the same letter are significantly different from one using Tukey’s test at 0.05 level.

**Table 6:** Overall time effects on sorghum grain weight loss during the 2014/15 and 2015/16 storage season in Mbire district, Zimbabwe (n = 56).

Storage time (weeks)	2014/15 season	2015/16 season
0	0.7 ± 0.10a	1.6 ± 0.08a
8	0.9 ± 0.13a	1.9 ± 0.17a
16	2.0 ± 0.15b	2.2 ± 0.20a
24	2.9 ± 0.35c	2.6 ± 0.26a
32	5.5 ± 0.82d	6.9 ± 0.75b
P-value	< .001	< .001
F-value	44.91	33.42

Means are compared per column, and those which do not share the same letter are significantly different from one another using Fishers Protected LSD test at 0.05 level.

### Insect population

**Figure 3:**
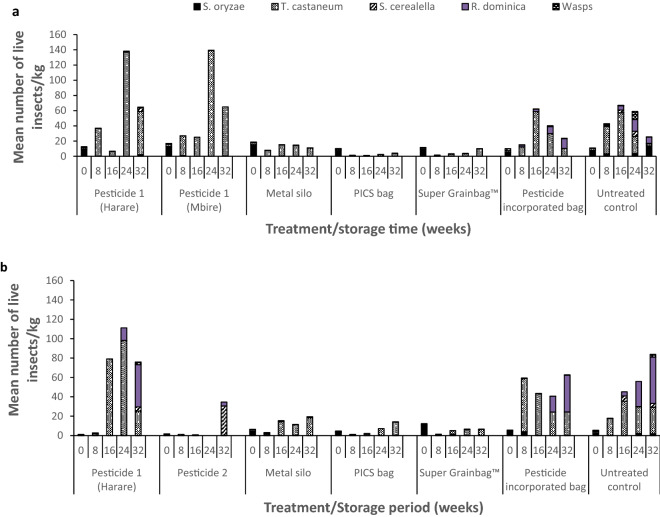
Mean total number of live storage insects per kg of sorghum grain sample in Mbire district, Zimbabwe during: (**a**) the 2014/15 and (**b**) the 2015/16 storage seasons (n = 8). Pesticide 1 = fenitrothion 1% + deltamethrin 0.13%; Pesticide 2 = pirimiphos-methyl 0.16% + thiamethoxam 0.036%; Pesticide-incorporated bag = deltamethrin-incorporated polypropylene bag at 3 mg/kg.

The dominant pest species in both seasons were *Tribolium castaneum* (Heibst) (Coleoptera: Tenebrionidae), *Rhyzopertha dominica* (Fabricius) (Coleoptera: Bostrichidae) and *Sitotroga cerealella* (Olivier) (Lepidoptera: Gelechiidae). However, only low numbers of live *S. cerealella* were recorded in the treatments over the two experiment seasons. The *T. castaneum* populations developed more in grain treated with Pesticide 1 containing fenitrothion and deltamethrin (Fig. 3a,b), whilst *S. cerealella* population build-up was more pronounced in Pesticide 2 (containing pirimiphos-methyl and thiamethoxam) and untreated control treatments. Population growth of the insect-pests *T. castaneum* and *R. dominica* were more pronounced in deltamethrin-incorporated polypropylene storage bags and untreated control. The insect population build-up started from four months of storage (16 weeks) in synthetic pesticide treated grain (Fig. 3a,b). *Sitophilus oryzae* failed to develop significantly during the course of the experiment. Live adult *S. oryzae* were found at 32 weeks storage (during the winter months of May), when mean monthly temperatures at the experimental site had dropped to ~ 30 °C.

The hermetic treatments (PICS bags, Super Grainbags and metal silos) suppressed pest build-up during the full length of the two storage seasons, but small *T. castaneum* populations managed to develop in some metal silos and PICS bags. Massive amounts of moth webbing were observed in the untreated control and Pesticide 2 but only low numbers of moths were actually recorded during sampling. Development of *T. castaneum* was relatively low in untreated control where *R. dominica* and *S. cerealella* populations developed (Fig. 3b).

Correlation analysis between individual insect species and grain damage and grain weight losses showed that live *R. dominica* had a medium positive correlation with grain damage (0.41 and 0.55) and grain weight loss (0.35 and 0.62) during the 2014/15 and 2015/16 storage seasons, respectively (Table 7). Total populations (dead and live) of *R.* *dominica* had medium to high positive correlation with grain damage (0.48 and 0.73), weight loss (0.37 and 0.70) during the 2014/15 and 2015/16 storage seasons, respectively (Table 8). The pest, *R.* *dominica* also had a moderate (0.37) and weak (0.13) positive correlation with grain moisture content during the first and second storage seasons, respectively. The number of live *S.* *cerealella* had a weak positive relationship with grain damage and grain weight loss, while live *T. castaneum* numbers had no relationship with the measured parameters (0.1 and 0.09 for grain damage and 0.16 and 0.13 for weight losses) in either season. However, total *T. castaneum* numbers (live and dead) had a relatively high correlation with grain moisture content (0.55) (Table 8). *Sitophilus oryzae* live insect counts also had very weak positive correlation with grain damage (0.05 and 0.12) and grain weight loss (0.01 and 0.08) during the 2014/15 and 2015/16 seasons, respectively (Table 7).

**Table 7:** Correlations between the number of live insects per species and percentage sorghum grain damage and weight loss during the 2014/15 and 2015/16 storage seasons (n = 280).

Insect species	Grain damage		Grain weight loss	
	2014/15	2015/16	2014/15	2015/16
*S. oryzae*	0.05	0.12	0.01	0.08
*T. castaneum*	0.10	0.09	0.16	0.13
*S. cerealella*	0.11	0.31	0.02	0.30
*R. dominica*	0.41	0.55	0.35	0.62

**Table 8:** Correlations between the total populations (live and dead) of each storage insect pest species present and sorghum grain damage, weight loss and grain moisture content during the 2014/15 and 2015/16 storage seasons (n = 280).

Insect species	2014/15 season			2015/16 season		
	Grain damage	Moisture content	Weight loss	Grain damage	Moisture content	Weight loss
*R. dominica*	0.48	0.23	0.37	0.73	0.13	0.7
*S. cerealella*	0.12	0.18	0.05	0.39	0.29	0.33
*S. oryzae*	0.04	0.07	0.02	0.05	0.03	0.03
*T. castaneum*	0.23	0.55	0.22	0.65	0.12	0.59

### Grain moisture content

**Figure 4:**
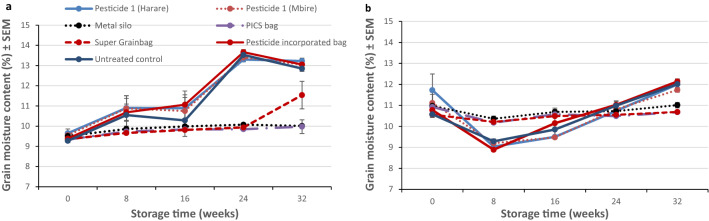
Mean sorghum grain moisture content (% ± SEM) in different storage treatments during: (**a**) the 2014/15 and (**b**) the 2015/16 storage seasons in Mbire district, Zimbabwe (n = 8). Pesticide 1 = fenitrothion 1% + deltamethrin 0.13%; Pesticide 2 = pirimiphos-methyl 0.16% + thiamethoxam 0.036%; Pesticide-incorporated bag = deltamethrin-incorporated polypropylene bag at 3 mg/kg.

As the storage duration increased, grain moisture content began to vary between the treatments under evaluation. The metal silo and the two hermetic bags maintained a constant grain moisture content throughout the two storage seasons (Fig. 4a,b). However, there was an increase in grain moisture content in the hermetic bag with a single plastic liner inside the polypropylene bag (Super Grainbag) at week 32 (from 10 to 12%) during the 2014/15 storage season. There were fluctuations in grain moisture content in all non-hermetic treatments (Fig. 4a,b) during the two storage seasons.

During the dry months in Mbire (September–November), grain moisture content dropped in all non-hermetic treatments, and then increased during the rainy periods, from week 16–32 (December–January) during the 2014/15 storage season and 24–32 (December–March) during the 2015/16 storage season. The initial grain moisture content was lower at set-up in the 2014/15 (~ 9.5%), than the 2015/16 season (~ 11%).

### Grain rotting

There were no significant differences in grain rotting between all the treatments used in either of the two seasons. However, initial percentage of rotten grains was higher in the second (2015/16) season than the first storage season (2014/15), but it remained below 4% throughout the two storage seasons.

### Ambient environmental conditions

**Figure 5:**
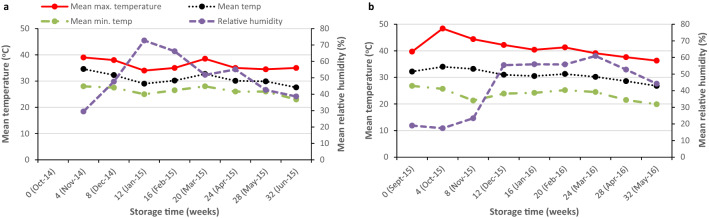
Ambient temperature and relative humidity conditions within the storage rooms in Mbire district, Zimbabwe during (**a**) the 2014/15 and (**b**) the 2015/16 storage seasons.

The mean temperatures within the storage facilities averaged between 25 and 30 °C over the two storage seasons, with high diurnal variations and temperatures rising up to 40 °C in both seasons. Relative humidity was generally low (≤ 50%) throughout the two seasons, except between 8 and 16 weeks during the first storage season when it rose up to ~ 70%. Temperature during the same period slightly dropped to a range of 25–35 °C. During the second storage season, relative humidity went as low as 20% during the first 8 weeks of the second season. Relative humidity rose to ~ 55% from 8 weeks of storage in the second season while temperatures dropped to an average of 30 °C for the rest of the storage season (Fig. 5).

## Discussion

### Grain damage and weight loss

Significant differences in percentage grain damage were found between treatments, with the hermetic storage technologies outperforming the non-hermetic treatments during the entire 32 week storage period in the two seasons, suggesting the containers remained relatively hermetic throughout the experiment. The respiration of the live insects and other live organisms within the hermetic storage technologies will have created hypoxic (low oxygen) and hypercarbic (high carbon dioxide) conditions which kill insects^18,22,34,35^. The gas-tight containers (hermetic storage technologies) retard the movement of oxygen into the stored grain from the outside environment, resulting in desiccation and death of insect pests as a result of shut down in the production of metabolic water. Oxygen transmission rate data for PICS bags were not available from the manufacturer, which prevented comparison of this aspect between the two hermetic bag brands tested which would be informative regards their potential efficacy. Additionally, gas composition within the storage containers was not measured during the study to determine whether the treatments remained hermetic throughout the study and to determine their level of hermeticity, therefore no discussion of the comparative gas retention can be provided. These data would have added more value to the scientific information obtained from the current study and enable comparison of the hermeticity of the individual containers under smallholder farmer-storage conditions. This is an aspect recommended for inclusion in future field studies.

When storage insects are exposed to hypoxic and hypercarbic conditions, they usually adapt by reducing their respiration and metabolic rates, and shifting to discontinuous gas exchange cycle where they can take relatively long periods with their spiracles fully closed, hence their desiccation is gradual^36–39^. In addition, terrestrial insects also lose water when they continually ventilate their tracheal linings when exposed to dry conditions^22^. The superior efficacy of the hermetic storage technologies as compared to the synthetic pesticides in suppressing insect damage in stored sorghum grain suggests they can be recommended and promoted for sorghum grain storage.

Significant differences between treatments with regards to grain weight loss were recorded in in the two storage seasons (p < 0.01). Weight losses were low in all the three hermetic storage technologies (metal silo and the two hermetic bags) (< 3%) and there were no significant differences between these three treatments. These results are similar to those of Baoua et al.^40^ who found no significant differences in pest populations and grain damage between PICS bags and Super Grainbags when compared side-by-side under laboratory environments in Niger. This means farmers can use any of the three hermetic containers tested, considering their availability and cost, and get the same level of efficacy in protecting their stored grain from insect damage.

The arrested insect pest development in the hermetic storage technologies resulted in low grain damage and grain weight loss levels while the synthetic pesticide treatments failed to suppress insect pest build-up, and experienced high grain weight loss due to insect feeding. These results suggest the insect pests may have built up resistance to the tested pesticides, the product quality was sub-standard, or that the high temperatures experienced in Mbire district led to reduced efficacy. Zimbabwe, like many other countries, relies on the use of a narrow range of synthetic pesticides, dominated by organophosphates and synthetic pyrethroids^11,41^. Continuous exposure of pests to the same active ingredients over time has implications for the development of pest resistance^42^. The suspected tolerance to a binary synthetic pesticide containing fenitrothion and deltamethrin (Pesticide 1) used in this study has also been found in a parallel study conducted in both Mbire and Harare during the same storage season using sorghum grain^43^. Poor performance by this pesticide was also recorded against maize storage insect pests in different parts of Zimbabwe^17,34^. The overall weight losses recorded in untreated grain (13.8 and 8.2%) and deltamethrin-incorporated polypropylene storage bags (10.8 and 10.5%) during the first and second season respectively are much higher than the country’s mean estimated sorghum grain storage weight losses of 2.3–3.0%, although consumption withdrawals were not factored into the weight loss figures in the trial ^8^.

### Storage insect dynamics

The dominant insect pests found in the sorghum stored in Mbire district during these experiments were *T. castaneum*, *R. dominica* and *S. cerealella*. The high positive correlation between grain moisture content and *R. dominica* and *T. castaneum* and *S. cerealella* suggests the insects were responsible for the increase in grain moisture content during storage. These findings are similar to those from a parallel study conducted in Mbire district and Harare, where a range of registered synthetic pesticides were tested for efficacy during the same storage season^43^. The results also concur to those of Stathers et al.^44^ where they identified the same storage insects as dominant in stored sorghum in Binga district, also located in the western side of the Zambezi Valley of Zimbabwe. However, in the current experiments, low populations of *S. cerealella* were recorded, most of which were dead. The sampling method used may have contributed to the low recorded numbers of this moth, as the highly mobile moth may have escaped entry into the sampling probes^45,46^. In addition, the sieving done during sample analyses can break up the delicate moth bodies, resulting in low *S. cerealella* counts. Subsequent incubation of the samples can help provide better estimates of delicate moth pest numbers. However, this was not done in the current experiments but should be a feature of any future sorghum storage studies.

Storage insect pest populations were high in the non-hermetic treatments (Pesticide 1, deltamethrin-incorporated polypropylene bags and untreated control). Failure of the pesticide containing 0.13% deltamethrin and 1% fenitrothion (locally-sourced and that obtained from a registered stockist) was also recorded against maize storage insect pests in Zimbabwe where the larger grain borer (*Prostephanus truncatus*) developed extensively in grain treated with this pesticide^34^. The dominant pest in the pesticide treated bags was *T. castaneum,* high populations of which developed in this pesticide. Similar to the findings in Mbire district, where a range of synthetic pesticides were evaluated on stored sorghum, *T.* *castaneum* was dominant in the Shumba Super dust treatment^43^. This could be a result of development of some tolerance or resistance to the pesticide. The pest also multiplied in the storage bags with deltamethrin incorporated into their fabric. Deltamethrin is also one of the active ingredients in Shumba Super dust. The newly introduced Pesticide 2 containing thiamethoxam and pirimiphos-methyl recorded low insect pest population build-up during the 2015/16 storage season, but the webbing of *S. cerealella* was evident. Insect pest resistance is a result of genetic selection over time and/or misuse of pesticides by farmers^14^. Some resistant strains of small grain storage pests have been reported in Brazil^47^, in particular *R. dominica* strains showing resistance to deltamethrin^48^ and pirimiphos-methyl^49^. More cases of resistance by storage insect pests have been reported by other researchers across the world^48–50^.

Insecticide-incorporated polypropylene storage bags are designed to kill insects which come into contact with the fabric of the bag^51^. Thus, any insects inside the bag can develop as long as they do not come into contact with the bag surfaces. Grain to be stored using these bags should be disinfested by fumigation first to make it free from all stages of insect pests^52^. However, fumigation is not recommended under smallholder storage conditions in Zimbabwe and most of SSA; hence the grain loaded in these bags was not fumigated prior to experimentation. This enabled the testing of the appropriateness of these bags under realistic smallholder management conditions. The insecticide-incorporated storage bags did not perform well when used with non-fumigated grain. Having recognised this problem, the company has recently developed and started promoting a hermetic bag with pesticide incorporated in the outer polypropylene bag.

The development of *T. castaneum* in metal silos can be attributed to possible loss of hermeticity due to the high temperatures in Mbire district which caused cracking and loosening of the elastic bands used to fasten the inlet and outlet valves of the silos. The results may also suggest that this pest is able to survive under low oxygen conditions, which warrants further investigation. Previous studies showed that *T. castaneum* mortality is low at oxygen levels above 4%^53,54^. The current study did not have gas monitoring equipment, or a pressure test to determine the oxygen-carbon dioxide levels in the containers, and gas permeability levels of the individual containers to link with *T. castaneum* response. This equipment could have helped determine precisely the oxygen-carbon dioxide levels under which these insects survived or the possible changes in pressure, pointing to possible loss of hermeticity. However, our results showing superiority of hermetic facilities compared to non-hermetic synthetic pesticide grain protectant treatments are similar to those found in stored cowpeas^36^, maize^17,34,55^ and sorghum^35^.

### Grain moisture content and rotting

The absence of significant differences between treatments, and over time, with regards to the number of rotten grains shows that despite the risk of mould formation and grain rotting due to moisture ingress in non-hermetic treatments, the moisture content fluctuations remained within safe storage levels. However, if the grain moisture content increases to above 13.5%, the grain may rot. Higher moisture content in stored grain increases the risks of mould development, development of heat patches and mycotoxin contamination from fungal activity^56^. This can be caused by rewetting, which is likely to occur in non-hermetic treatments in response to fluctuations in the outside environment. The maintenance of constant grain moisture content conditions by hermetic storage technologies make them useful for long-term grain storage as they can protect stored grain against the outside environment. These results are similar to those found in laboratory studies by Williams et al.^35^ when sorghum was stored using PICS bags and some other hermetic plastic containers.

The sharp drop and subsequent rise in grain moisture content in the non-hermetic treatments during the second storage season (between 0 and 16 weeks of storage) is attributed to high temperatures and low atmospheric moisture, marking the occurrence of mid-season dry-spells. Such spells are common in many parts of Zimbabwe during the rainy seasons. Other studies also found that hermetic treatments maintained constant moisture content of stored grain for periods of up to 12 months in maize^17,19,57^, sorghum^35^ and groundnuts^58^.

Although the treatments were housed in grass-thatched granaries to protect them from direct sunlight, and temperatures within the storage rooms were measured, measuring the temperature within each storage container could have provided some useful information regarding insect survival and whether some of the containers influence the temperatures within the enclosures. We therefore recommend that future work includes temperature and humidity data loggers within the storage containers in addition to equipment for measuring oxygen-carbon dioxide compositions.

### Access to and usability of the technologies by smallholder farmers

Despite the clear evidence of efficacy of the hermetic storage technologies during the two storage seasons, there are currently very limited institutional arrangements in place to enable interested farmers to purchase these technologies in Zimbabwe. For metal silos, the major limitation to the dissemination of these storage containers is their cost of production and transportation, which is beyond the reach of many smallholder farmers in the short-term^25^. Although the metal silos can be manufactured by anyone due to the absence of patents, the required materials are not easily accessible, labour demands and transportation costs are high^25^, making the final product unaffordable for a once-off payment by many smallholder farmers. For hermetic bags, many African countries rely on imports, which can make them too expensive for most subsistence farmers. However, the hermetic bag technologies can be affordable and sustainable in the long-term if manufactured in-country as is already the case in a few countries in SSA, e.g. Kenya, Malawi, Tanzania and Zambia. Research has demonstrated that if the bags are bored by insects or rodents, they can be patched (using glue and extra inner lining) to maintain the hermeticity and continue to be effective in storing grain; hence they can be used for more than one season^59^. There are no other published studies that have focused on hermetic storage of sorghum grain and its associated pests under typical smallholder conditions and involving stakeholders. Evaluation of these technologies by multiple stakeholders can help raise awareness, develop demand, aid in improving distribution of the effective options. Multi-stakeholder testing can be easily combined with training on the proper use of the technologies, and possibly increase their uptake and adoption. Recent evidence synthesis of small-scale farmer postharvest loss reduction technologies in SSA and South Asia revealed that there are very few studies which involve the end-users in the testing of the technologies, or conduct multi-site or multi-year comparisons of the interventions^10^.

## Conclusions

The hermetic storage technologies performed significantly better than the synthetic pesticides in protecting stored sorghum grain from insect damage, which provides scope for promotion of sorghum storage in hot and dry areas. These findings have important food security implications given the expected increased production of the more climate-resilient small grains such as sorghum by smallholder farmers in response to climatic changes, and the need to protect these grains from storage insect damage after harvest. However, before recommending these technologies for up-scaling, the re-usability of the hermetic bags should be tested to more accurately determine their durability, life-span and cost-efficacy under smallholder storage conditions and management practices as some of the bags were found to have been damaged by rodents, leading to the loss of hermeticity.

Results from these field experiments indicate the failure of the pesticide dust containing 0.13% deltamethrin and 1% fenitrothion, regardless of whether it was bought from a local agro-dealer shop or from a registered stockist in the capital city. *Tribolium castaneum* showed high levels of tolerance to the same pesticide, and to the deltamethrin-incorporated storage bag, and also in the supposedly low oxygen levels in metal silos. Populations of *S. cerealella* managed to develop in the grain treated with the newly introduced synthetic pesticide dust containing pirimiphos methyl and thiamethoxam. Analysis of the pesticide residues remaining on the grain at different time periods during the experiment would provide deeper understanding of the persistence of the pesticide under field conditions.

## Data Availability

The datasets generated during and/or analysed during the current study are available from the corresponding author on reasonable request.
